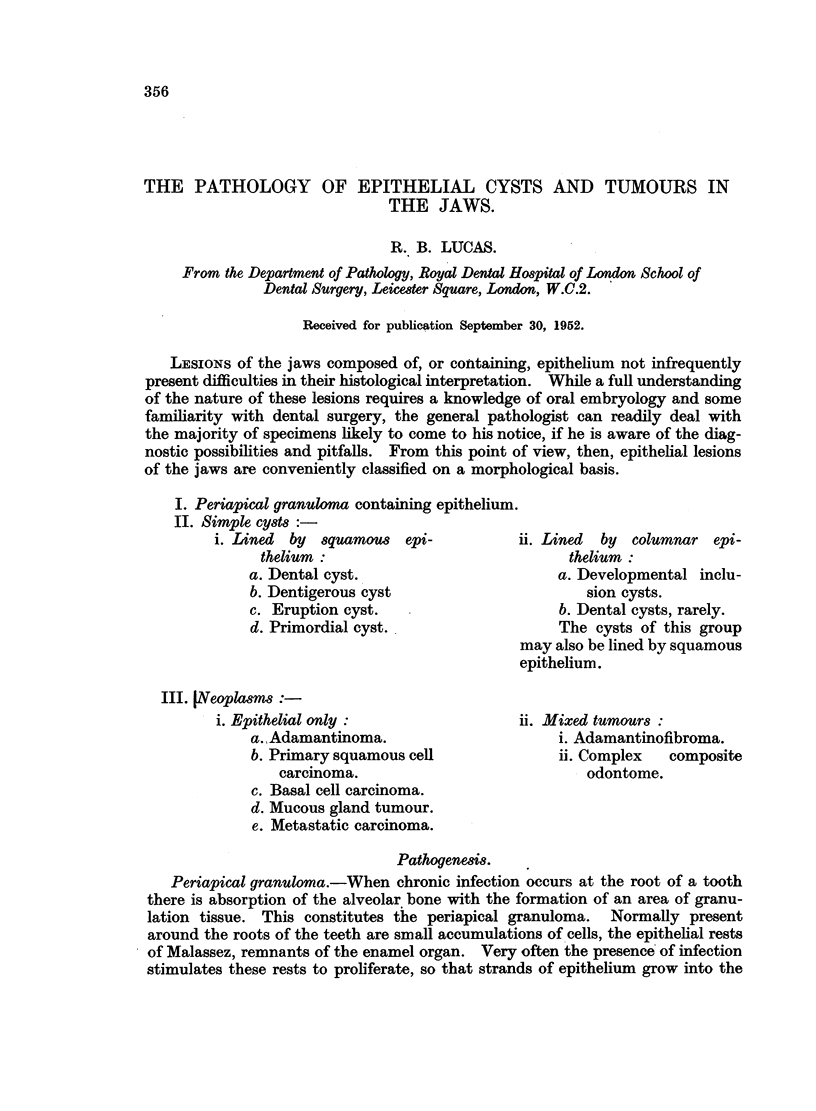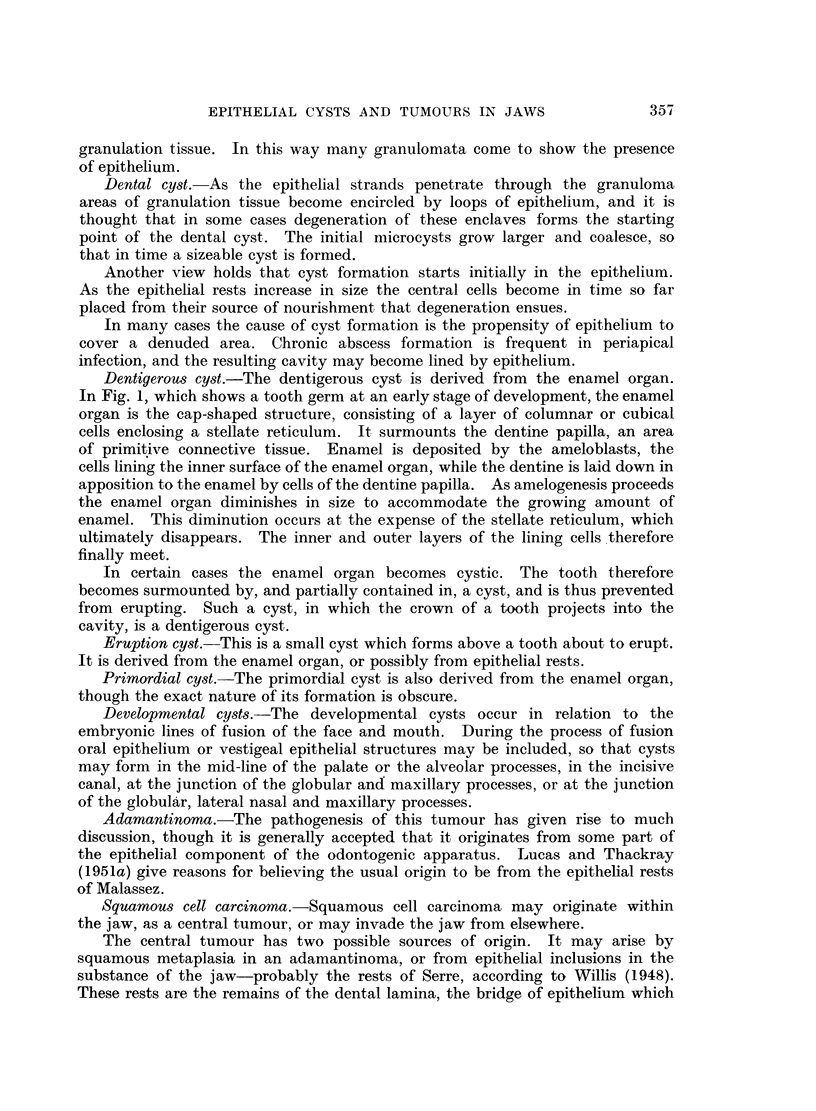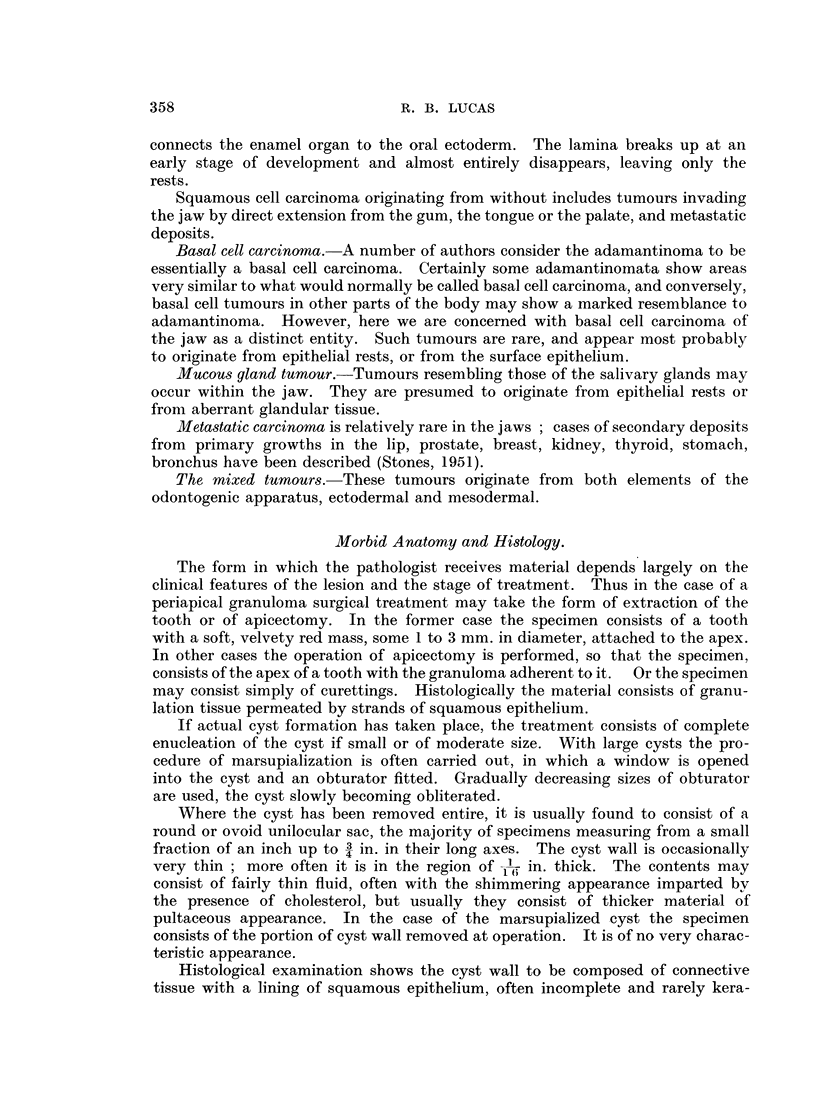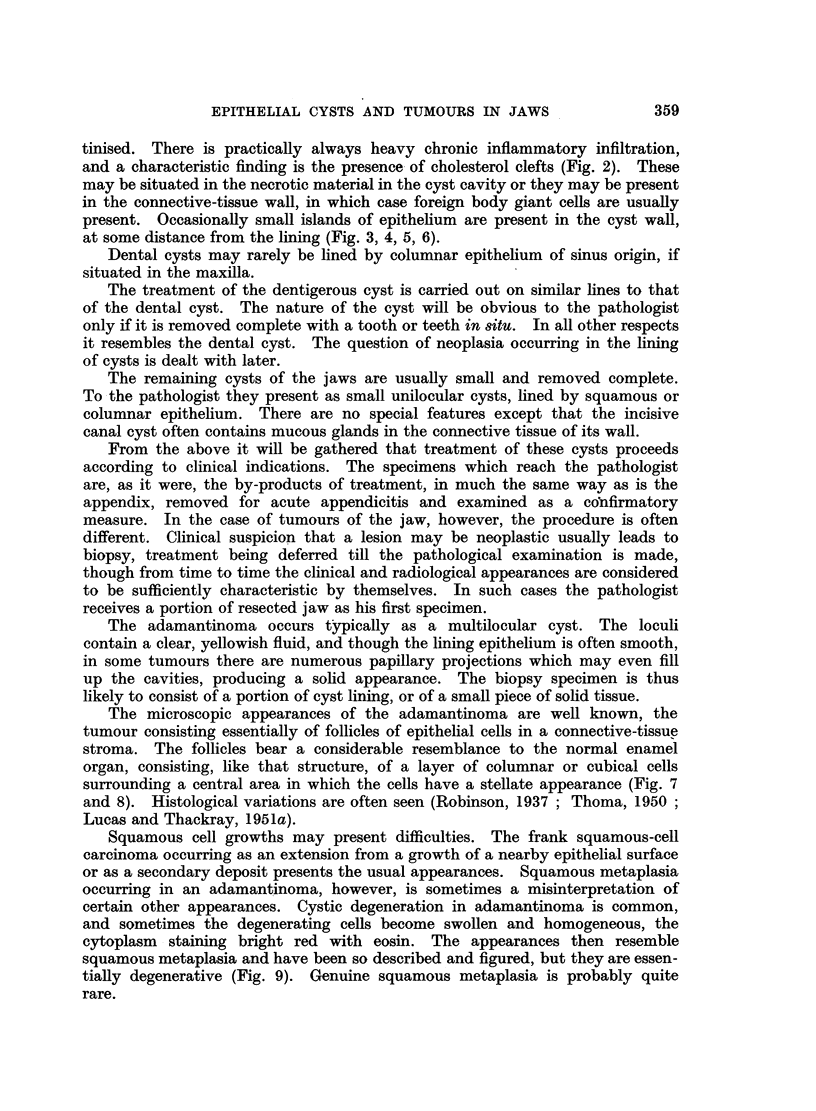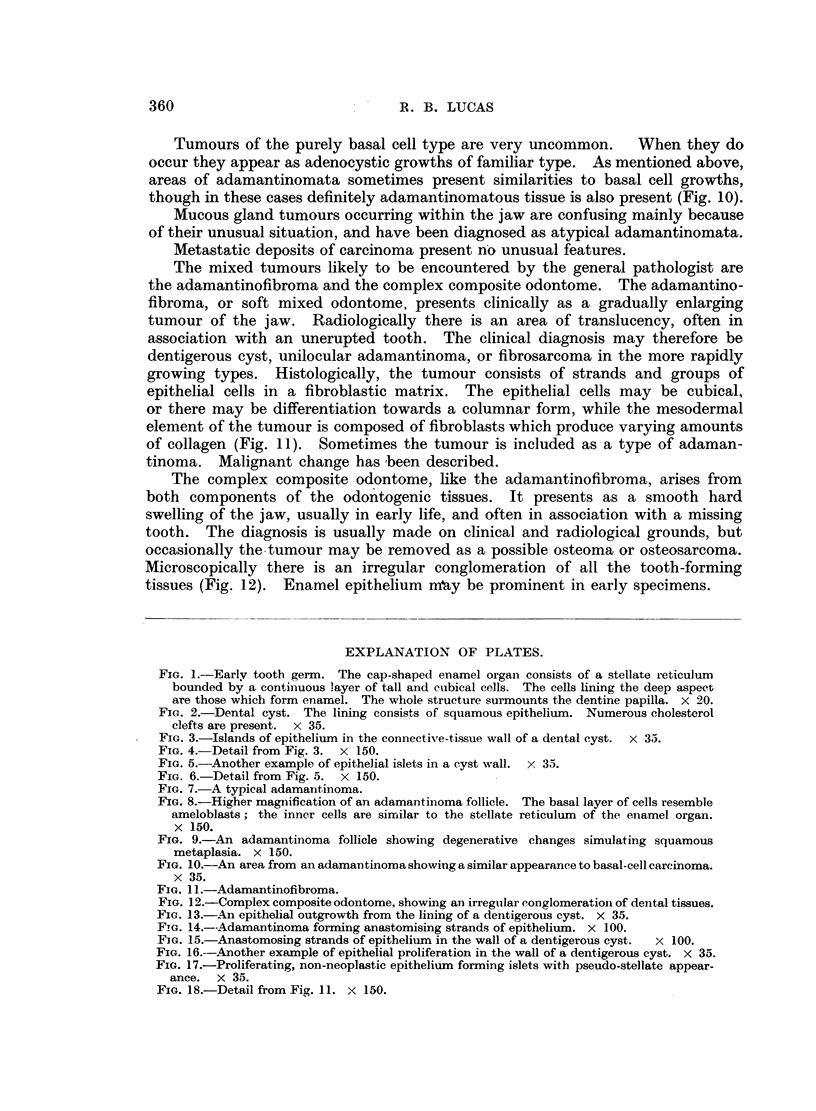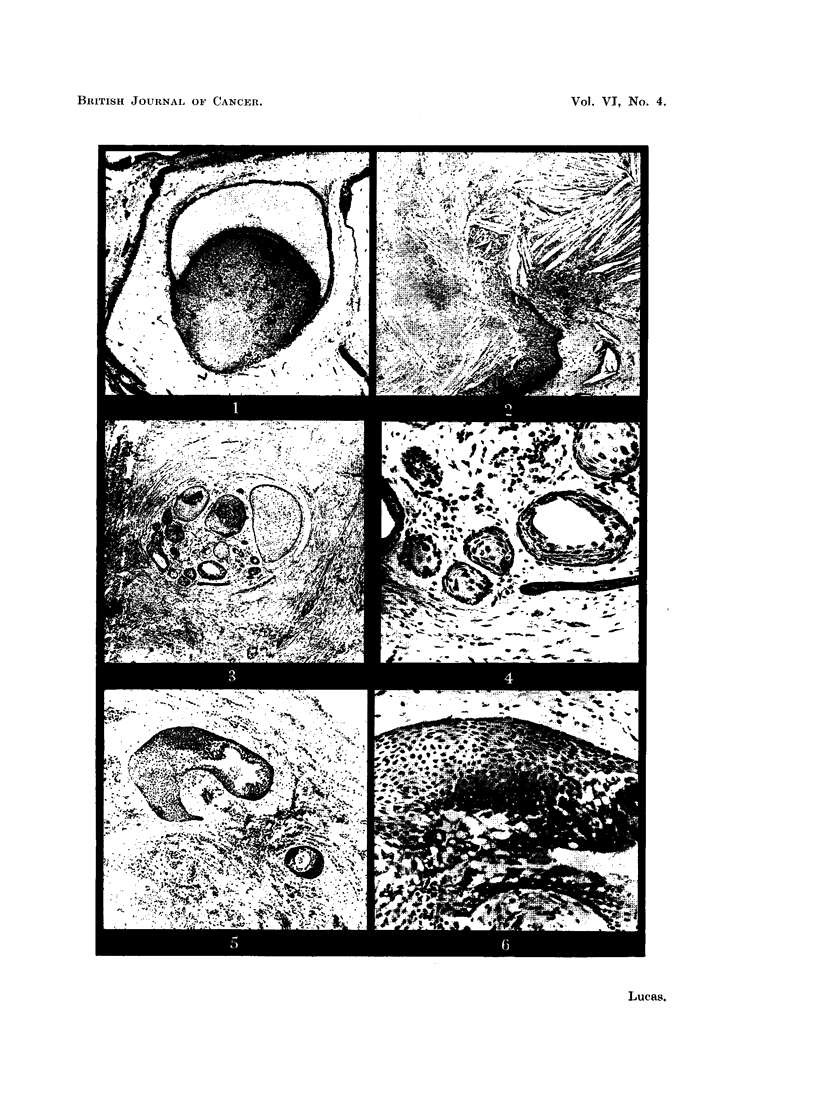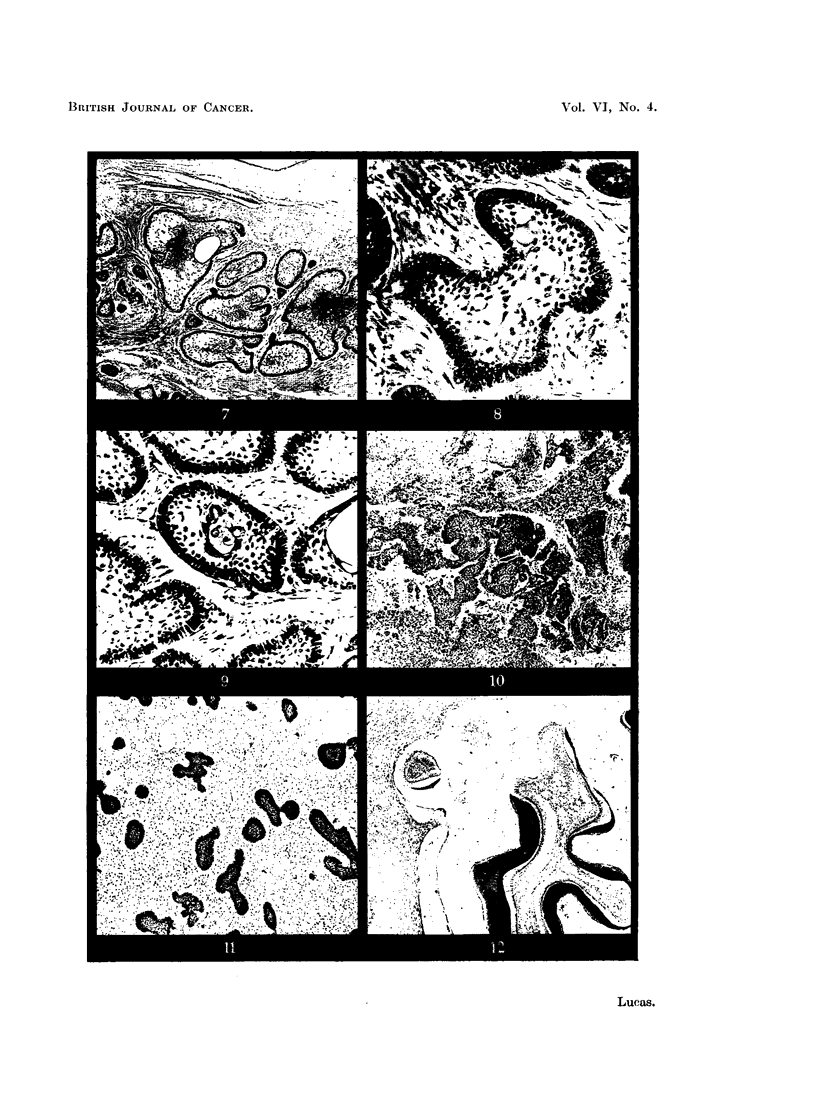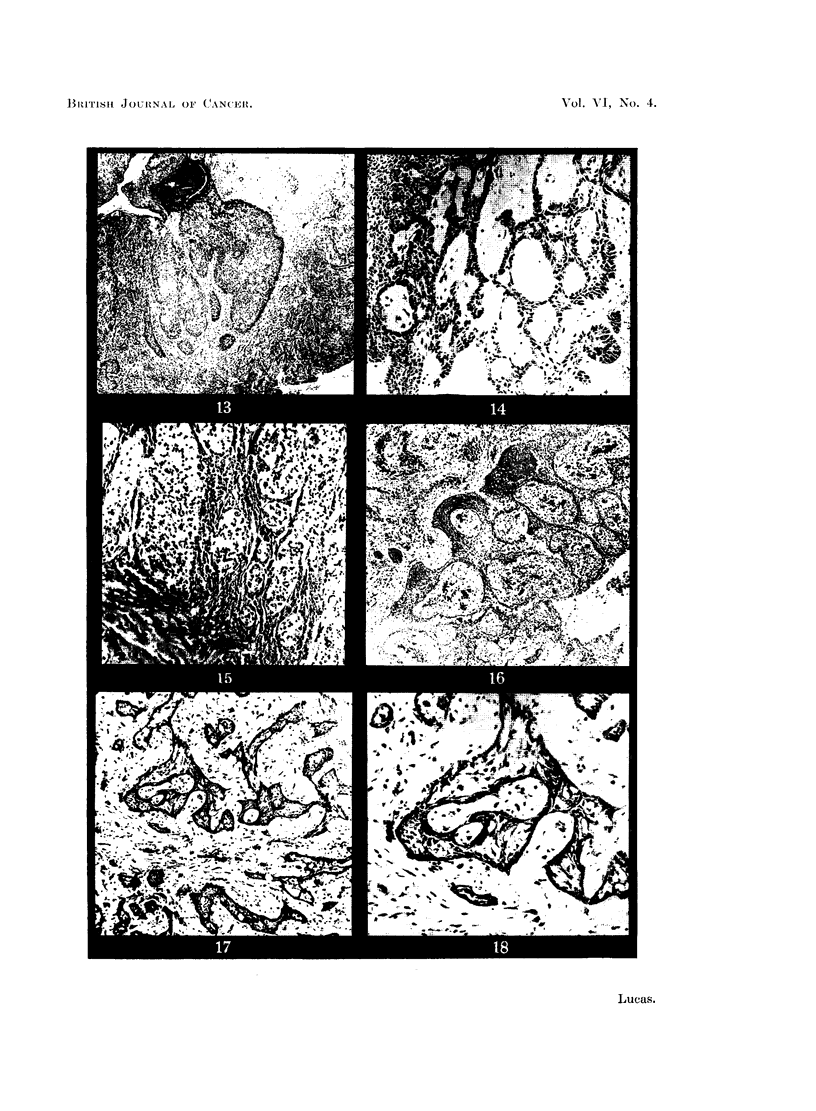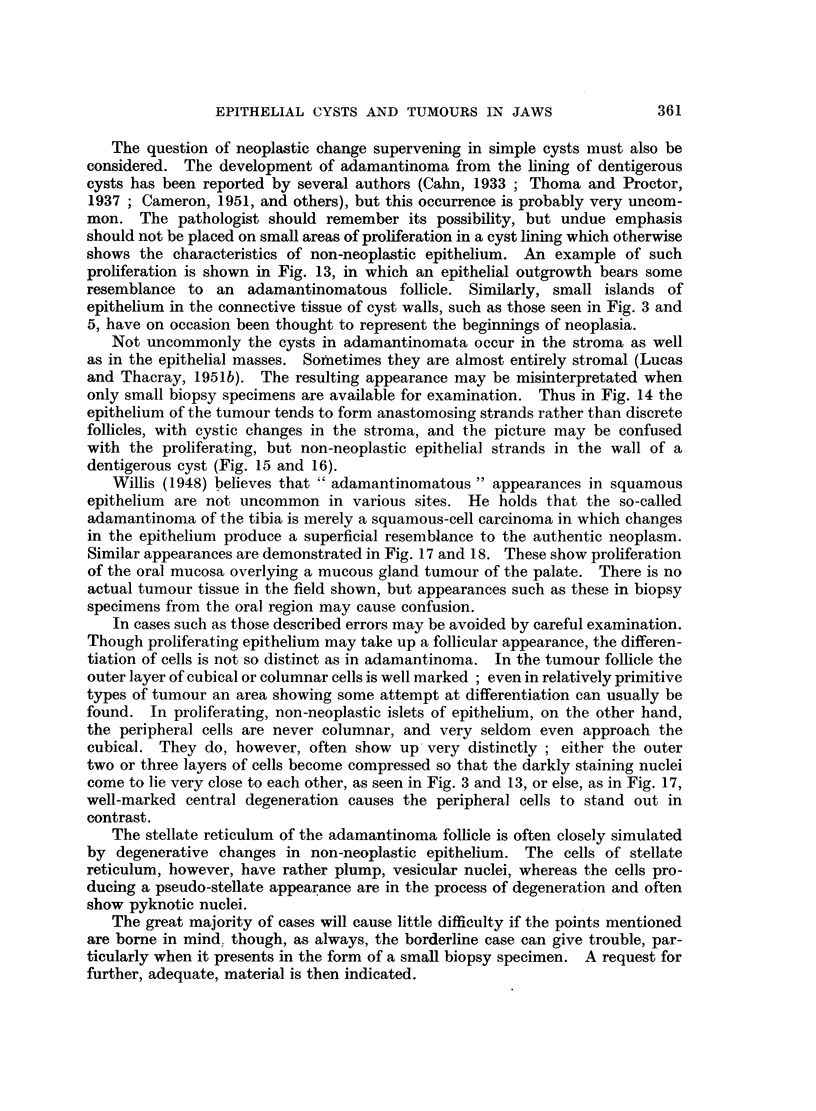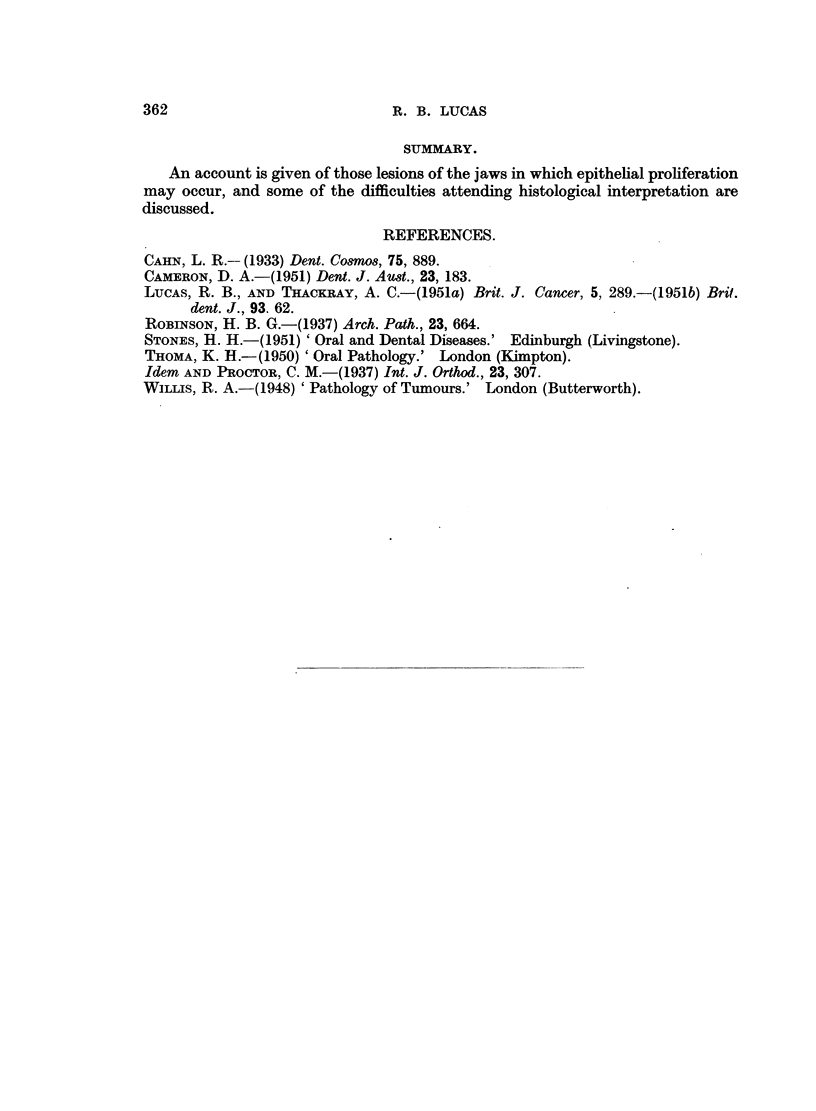# The Pathology of Epithelial Cysts and Tumours in the Jaws

**DOI:** 10.1038/bjc.1952.39

**Published:** 1952-12

**Authors:** R. B. Lucas

## Abstract

**Images:**


### THE PATHOLOGY OF EPITHELIAL CYSTS AND TUMOURS IN THE JAWS.

R. B. LUCAS.

*From the Department of Pathology, Royal Dental Hospital of London School of Dental Surgery, Leicester Square, London, W.C.2.*

Received for publication September 30, 1952.

LESIONS of the jaws composed of, or containing, epithelium not infrequently present difficulties in their histological interpretation. While a full understanding of the nature of these lesions requires a knowledge of oral embryology and some familiarity with dental surgery, the general pathologist can readily deal with the majority of specimens likely to come to his notice, if he is aware of the diagnostic possibilities and pitfalls. From this point of view, then, epithelial lesions of the jaws are conveniently classified on a morphological basis.

I. *Periapical granuloma* containing epithelium.

II. *Simple cysts* :—

- i. *Lined by squamous epithelium* :

  - *a*. Dental cyst.

  - *b*. Dentigerous cyst

  - *c*. Eruption cyst.

  - *d*. Primordial cyst.

- ii. *Lined by columnar epithelium* :

  - *a*. Developmental inclusion cysts.

  - *b*. Dental cysts, rarely.

  The cysts of this group may also be lined by squamous epithelium.

III. *Neoplasms* :—

- i. *Epithelial only* :

  - *a*. Adamantinoma.

  - *b*. Primary squamous cell carcinoma.

  - *c*. Basal cell carcinoma.

  - *d*. Mucous gland tumour.

  - *e*. Metastatic carcinoma.

- ii. *Mixed tumours* :

  - i. Adamantinofibroma.

  - ii. Complex composite odontome.

### *Pathogenesis.*

*Periapical granuloma.*—When chronic infection occurs at the root of a tooth there is absorption of the alveolar bone with the formation of an area of granulation tissue. This constitutes the periapical granuloma. Normally present around the roots of the teeth are small accumulations of cells, the epithelial rests of Malassez, remnants of the enamel organ. Very often the presence of infection stimulates these rests to proliferate, so that strands of epithelium grow into the granulation tissue. In this way many granulomata come to show the presence of epithelium.

*Dental cyst.*—As the epithelial strands penetrate through the granuloma areas of granulation tissue become encircled by loops of epithelium, and it is thought that in some cases degeneration of these enclaves forms the starting point of the dental cyst. The initial microcysts grow larger and coalesce, so that in time a sizeable cyst is formed.

Another view holds that cyst formation starts initially in the epithelium. As the epithelial rests increase in size the central cells become in time so far placed from their source of nourishment that degeneration ensues.

In many cases the cause of cyst formation is the propensity of epithelium to cover a denuded area. Chronic abscess formation is frequent in periapical infection, and the resulting cavity may become lined by epithelium.

*Dentigerous cyst.*—The dentigerous cyst is derived from the enamel organ. In Fig. 1, which shows a tooth germ at an early stage of development, the enamel organ is the cap-shaped structure, consisting of a layer of columnar or cubical cells enclosing a stellate reticulum. It surmounts the dentine papilla, an area of primitive connective tissue. Enamel is deposited by the ameloblasts, the cells lining the inner surface of the enamel organ, while the dentine is laid down in apposition to the enamel by cells of the dentine papilla. As amelogenesis proceeds the enamel organ diminishes in size to accommodate the growing amount of enamel. This diminution occurs at the expense of the stellate reticulum, which ultimately disappears. The inner and outer layers of the lining cells therefore finally meet.

In certain cases the enamel organ becomes cystic. The tooth therefore becomes surmounted by, and partially contained in, a cyst, and is thus prevented from erupting. Such a cyst, in which the crown of a tooth projects into the cavity, is a dentigerous cyst.

*Eruption cyst.*—This is a small cyst which forms above a tooth about to erupt. It is derived from the enamel organ, or possibly from epithelial rests.

*Primordial cyst.*—The primordial cyst is also derived from the enamel organ, though the exact nature of its formation is obscure.

*Developmental cysts.*—The developmental cysts occur in relation to the embryonic lines of fusion of the face and mouth. During the process of fusion oral epithelium or vestigeal epithelial structures may be included, so that cysts may form in the mid-line of the palate or the alveolar processes, in the incisive canal, at the junction of the globular and maxillary processes, or at the junction of the globular, lateral nasal and maxillary processes.

*Adamantinoma.*—The pathogenesis of this tumour has given rise to much discussion, though it is generally accepted that it originates from some part of the epithelial component of the odontogenic apparatus. Lucas and Thackray (1951*a*) give reasons for believing the usual origin to be from the epithelial rests of Malassez.

*Squamous cell carcinoma.*—Squamous cell carcinoma may originate within the jaw, as a central tumour, or may invade the jaw from elsewhere.

The central tumour has two possible sources of origin. It may arise by squamous metaplasia in an adamantinoma, or from epithelial inclusions in the substance of the jaw—probably the rests of Serre, according to Willis (1948). These rests are the remains of the dental lamina, the bridge of epithelium which connects the enamel organ to the oral ectoderm. The lamina breaks up at an early stage of development and almost entirely disappears, leaving only the rests.

Squamous cell carcinoma originating from without includes tumours invading the jaw by direct extension from the gum, the tongue or the palate, and metastatic deposits.

*Basal cell carcinoma.*—A number of authors consider the adamantinoma to be essentially a basal cell carcinoma. Certainly some adamantinomata show areas very similar to what would normally be called basal cell carcinoma, and conversely, basal cell tumours in other parts of the body may show a marked resemblance to adamantinoma. However, here we are concerned with basal cell carcinoma of the jaw as a distinct entity. Such tumours are rare, and appear most probably to originate from epithelial rests, or from the surface epithelium.

*Mucous gland tumour.*—Tumours resembling those of the salivary glands may occur within the jaw. They are presumed to originate from epithelial rests or from aberrant glandular tissue.

*Metastatic carcinoma* is relatively rare in the jaws ; cases of secondary deposits from primary growths in the lip, prostate, breast, kidney, thyroid, stomach, bronchus have been described (Stones, 1951).

*The mixed tumours.*—These tumours originate from both elements of the odontogenic apparatus, ectodermal and mesodermal.

### *Morbid Anatomy and Histology.*

The form in which the pathologist receives material depends largely on the clinical features of the lesion and the stage of treatment. Thus in the case of a periapical granuloma surgical treatment may take the form of extraction of the tooth or of apicectomy. In the former case the specimen consists of a tooth with a soft, velvety red mass, some 1 to 3 mm. in diameter, attached to the apex. In other cases the operation of apicectomy is performed, so that the specimen, consists of the apex of a tooth with the granuloma adherent to it. Or the specimen may consist simply of curettings. Histologically the material consists of granulation tissue permeated by strands of squamous epithelium.

If actual cyst formation has taken place, the treatment consists of complete enucleation of the cyst if small or of moderate size. With large cysts the procedure of marsupialization is often carried out, in which a window is opened into the cyst and an obturator fitted. Gradually decreasing sizes of obturator are used, the cyst slowly becoming obliterated.

Where the cyst has been removed entire, it is usually found to consist of a round or ovoid unilocular sac, the majority of specimens measuring from a small fraction of an inch up to $\frac{3}{4}$ in. in their long axes. The cyst wall is occasionally very thin ; more often it is in the region of $\frac{1}{16}$ in. thick. The contents may consist of fairly thin fluid, often with the shimmering appearance imparted by the presence of cholesterol, but usually they consist of thicker material of pultaceous appearance. In the case of the marsupialized cyst the specimen consists of the portion of cyst wall removed at operation. It is of no very characteristic appearance.

Histological examination shows the cyst wall to be composed of connective tissue with a lining of squamous epithelium, often incomplete and rarely keratinised. There is practically always heavy chronic inflammatory infiltration, and a characteristic finding is the presence of cholesterol clefts (Fig. 2). These may be situated in the necrotic material in the cyst cavity or they may be present in the connective-tissue wall, in which case foreign body giant cells are usually present. Occasionally small islands of epithelium are present in the cyst wall, at some distance from the lining (Fig. 3, 4, 5, 6).

Dental cysts may rarely be lined by columnar epithelium of sinus origin, if situated in the maxilla.

The treatment of the dentigerous cyst is carried out on similar lines to that of the dental cyst. The nature of the cyst will be obvious to the pathologist only if it is removed complete with a tooth or teeth *in situ*. In all other respects it resembles the dental cyst. The question of neoplasia occurring in the lining of cysts is dealt with later.

The remaining cysts of the jaws are usually small and removed complete. To the pathologist they present as small unilocular cysts, lined by squamous or columnar epithelium. There are no special features except that the incisive canal cyst often contains mucous glands in the connective tissue of its wall.

From the above it will be gathered that treatment of these cysts proceeds according to clinical indications. The specimens which reach the pathologist are, as it were, the by-products of treatment, in much the same way as is the appendix, removed for acute appendicitis and examined as a confirmatory measure. In the case of tumours of the jaw, however, the procedure is often different. Clinical suspicion that a lesion may be neoplastic usually leads to biopsy, treatment being deferred till the pathological examination is made, though from time to time the clinical and radiological appearances are considered to be sufficiently characteristic by themselves. In such cases the pathologist receives a portion of resected jaw as his first specimen.

The adamantinoma occurs typically as a multilocular cyst. The loculi contain a clear, yellowish fluid, and though the lining epithelium is often smooth, in some tumours there are numerous papillary projections which may even fill up the cavities, producing a solid appearance. The biopsy specimen is thus likely to consist of a portion of cyst lining, or of a small piece of solid tissue.

The microscopic appearances of the adamantinoma are well known, the tumour consisting essentially of follicles of epithelial cells in a connective-tissue stroma. The follicles bear a considerable resemblance to the normal enamel organ, consisting, like that structure, of a layer of columnar or cubical cells surrounding a central area in which the cells have a stellate appearance (Fig. 7 and 8). Histological variations are often seen (Robinson, 1937 ; Thoma, 1950 ; Lucas and Thackray, 1951*a*).

Squamous cell growths may present difficulties. The frank squamous-cell carcinoma occurring as an extension from a growth of a nearby epithelial surface or as a secondary deposit presents the usual appearances. Squamous metaplasia occurring in an adamantinoma, however, is sometimes a misinterpretation of certain other appearances. Cystic degeneration in adamantinoma is common, and sometimes the degenerating cells become swollen and homogeneous, the cytoplasm staining bright red with eosin. The appearances then resemble squamous metaplasia and have been so described and figured, but they are essentially degenerative (Fig. 9). Genuine squamous metaplasia is probably quite rare.

Tumours of the purely basal cell type are very uncommon. When they do occur they appear as adenocystic growths of familiar type. As mentioned above, areas of adamantinomata sometimes present similarities to basal cell growths, though in these cases definitely adamantinomatous tissue is also present (Fig. 10).

Mucous gland tumours occurring within the jaw are confusing mainly because of their unusual situation, and have been diagnosed as atypical adamantinomata.

Metastatic deposits of carcinoma present no unusual features.

The mixed tumours likely to be encountered by the general pathologist are the adamantinofibroma and the complex composite odontome. The adamantinofibroma, or soft mixed odontome, presents clinically as a gradually enlarging tumour of the jaw. Radiologically there is an area of translucency, often in association with an unerupted tooth. The clinical diagnosis may therefore be dentigerous cyst, unilocular adamantinoma, or fibrosarcoma in the more rapidly growing types. Histologically, the tumour consists of strands and groups of epithelial cells in a fibroblastic matrix. The epithelial cells may be cubical, or there may be differentiation towards a columnar form, while the mesodermal element of the tumour is composed of fibroblasts which produce varying amounts of collagen (Fig. 11). Sometimes the tumour is included as a type of adamantinoma. Malignant change has been described.

The complex composite odontome, like the adamantinofibroma, arises from both components of the odontogenic tissues. It presents as a smooth hard swelling of the jaw, usually in early life, and often in association with a missing tooth. The diagnosis is usually made on clinical and radiological grounds, but occasionally the tumour may be removed as a possible osteoma or osteosarcoma. Microscopically there is an irregular conglomeration of all the tooth-forming tissues (Fig. 12). Enamel epithelium may be prominent in early specimens.

---

EXPLANATION OF PLATES.

FIG. 1.—Early tooth germ. The cap-shaped enamel organ consists of a stellate reticulum bounded by a continuous layer of tall and cubical cells. The cells lining the deep aspect are those which form enamel. The whole structure surmounts the dentine papilla. × 20.

FIG. 2.—Dental cyst. The lining consists of squamous epithelium. Numerous cholesterol clefts are present. × 35.

FIG. 3.—Islands of epithelium in the connective-tissue wall of a dental cyst. × 35.

FIG. 4.—Detail from Fig. 3. × 150.

FIG. 5.—Another example of epithelial islets in a cyst wall. × 35.

FIG. 6.—Detail from Fig. 5. × 150.

FIG. 7.—A typical adamantinoma.

FIG. 8.—Higher magnification of an adamantinoma follicle. The basal layer of cells resemble ameloblasts; the inner cells are similar to the stellate reticulum of the enamel organ. × 150.

FIG. 9.—An adamantinoma follicle showing degenerative changes simulating squamous metaplasia. × 150.

FIG. 10.—An area from an adamantinoma showing a similar appearance to basal-cell carcinoma. × 35.

FIG. 11.—Adamantinofibroma.

FIG. 12.—Complex composite odontome, showing an irregular conglomeration of dental tissues.

FIG. 13.—An epithelial outgrowth from the lining of a dentigerous cyst. × 35.

FIG. 14.—Adamantinoma forming anastomising strands of epithelium. × 100.

FIG. 15.—Anastomosing strands of epithelium in the wall of a dentigerous cyst. × 100.

FIG. 16.—Another example of epithelial proliferation in the wall of a dentigerous cyst. × 35.

FIG. 17.—Proliferating, non-neoplastic epithelium forming islets with pseudo-stellate appearance. × 35.

FIG. 18.—Detail from Fig. 11. × 150.

Lucas.

Lucas.

13

14

15

16

17

18

The question of neoplastic change supervening in simple cysts must also be considered. The development of adamantinoma from the lining of dentigerous cysts has been reported by several authors (Cahn, 1933 ; Thoma and Proctor, 1937 ; Cameron, 1951, and others), but this occurrence is probably very uncommon. The pathologist should remember its possibility, but undue emphasis should not be placed on small areas of proliferation in a cyst lining which otherwise shows the characteristics of non-neoplastic epithelium. An example of such proliferation is shown in Fig. 13, in which an epithelial outgrowth bears some resemblance to an adamantinomatous follicle. Similarly, small islands of epithelium in the connective tissue of cyst walls, such as those seen in Fig. 3 and 5, have on occasion been thought to represent the beginnings of neoplasia.

Not uncommonly the cysts in adamantinomata occur in the stroma as well as in the epithelial masses. Sometimes they are almost entirely stromal (Lucas and Thacray, 1951*b*). The resulting appearance may be misinterpretated when only small biopsy specimens are available for examination. Thus in Fig. 14 the epithelium of the tumour tends to form anastomosing strands rather than discrete follicles, with cystic changes in the stroma, and the picture may be confused with the proliferating, but non-neoplastic epithelial strands in the wall of a dentigerous cyst (Fig. 15 and 16).

Willis (1948) believes that " adamantinomatous " appearances in squamous epithelium are not uncommon in various sites. He holds that the so-called adamantinoma of the tibia is merely a squamous-cell carcinoma in which changes in the epithelium produce a superficial resemblance to the authentic neoplasm. Similar appearances are demonstrated in Fig. 17 and 18. These show proliferation of the oral mucosa overlying a mucous gland tumour of the palate. There is no actual tumour tissue in the field shown, but appearances such as these in biopsy specimens from the oral region may cause confusion.

In cases such as those described errors may be avoided by careful examination. Though proliferating epithelium may take up a follicular appearance, the differentiation of cells is not so distinct as in adamantinoma. In the tumour follicle the outer layer of cubical or columnar cells is well marked ; even in relatively primitive types of tumour an area showing some attempt at differentiation can usually be found. In proliferating, non-neoplastic islets of epithelium, on the other hand, the peripheral cells are never columnar, and very seldom even approach the cubical. They do, however, often show up very distinctly ; either the outer two or three layers of cells become compressed so that the darkly staining nuclei come to lie very close to each other, as seen in Fig. 3 and 13, or else, as in Fig. 17, well-marked central degeneration causes the peripheral cells to stand out in contrast.

The stellate reticulum of the adamantinoma follicle is often closely simulated by degenerative changes in non-neoplastic epithelium. The cells of stellate reticulum, however, have rather plump, vesicular nuclei, whereas the cells producing a pseudo-stellate appearance are in the process of degeneration and often show pyknotic nuclei.

The great majority of cases will cause little difficulty if the points mentioned are borne in mind, though, as always, the borderline case can give trouble, particularly when it presents in the form of a small biopsy specimen. A request for further, adequate, material is then indicated.

SUMMARY.

An account is given of those lesions of the jaws in which epithelial proliferation may occur, and some of the difficulties attending histological interpretation are discussed.

REFERENCES.

Cahn, L. R.—(1933) *Dent. Cosmos*, **75**, 889.
Cameron, D. A.—(1951) *Dent. J. Aust.*, **23**, 183.
Lucas, R. B., and Thackray, A. C.—(1951*a*) *Brit. J. Cancer*, **5**, 289.—(1951*b*) *Brit. dent. J.*, **93**. 62.
Robinson, H. B. G.—(1937) *Arch. Path.*, **23**, 664.
Stones, H. H.—(1951) 'Oral and Dental Diseases.' Edinburgh (Livingstone).
Thoma, K. H.—(1950) 'Oral Pathology.' London (Kimpton).
*Idem* and Proctor, C. M.—(1937) *Int. J. Orthod.*, **23**, 307.
Willis, R. A.—(1948) 'Pathology of Tumours.' London (Butterworth).